# Trace determination of lenalidomide in plasma by non-extractive HPLC procedures with fluorescence detection after pre-column derivatization with fluorescamine

**DOI:** 10.1186/1752-153X-7-52

**Published:** 2013-03-14

**Authors:** Nasr Y Khalil, Ibrahim A Darwish, Tanveer A Wani, Abdel-Rahman A Al-Majed

**Affiliations:** 1 Department of Pharmaceutical Chemistry, College of Pharmacy, King Saud University, P.O. Box 2457, Riyadh, 11451, Saudi Arabia

**Keywords:** Lenalidomide, Fluorescamine, HPLC, Fluorescence detection, Therapeutic drug, Monitoring, Pharmacokinetic studies

## Abstract

**Background:**

Lenalidomide (LND) is a new potent drug used for treatment of multiple myeloma. For its pharmacokinetic studies and therapeutic monitoring, a proper analytical method was required.

**Results:**

In this study, a non extractive and simple pre-column derivatization procedures have been proposed, for the for trace determination of lenalidomide (LND) in human plasma by HPLC with fluorescence detection. Plasma samples were treated with acetonitrile for protein precipitation then treated with copper acetate to form stable complexes with the biogenic amines and mask their interference with the derivatization reaction of LND. Treated plasma samples containing LND was derivatized with fluorescamine (FLC) in aqueous media at ambient temperature. Separation of the derivatized LND was performed on Hypersil BDS C18 column (250 × 4.6 mm, 5 μm particle size) using a mobile phase consisting of phosphate buffer (pH 4):methanol: tetrahydrofuran (70:10:20, v/v) at a flow rate of 1.0 mL/min. The derivatized samples were monitored at an emission wavelength of 495 nm after excitation at a wavelength of 382 nm. Under the optimum chromatographic conditions, a linear relationship with good correlation coefficient (r = 0.9997, n = 9) was found between the peak area and LND concentrations in the range of 2–100 ng/mL. The limits of detection and quantitation were 0.8 and 2.30 ng/mL, respectively. The intra- and inter-assay precisions were satisfactory and the accuracy of the method was proved. The recovery of LND from the spiked human plasma was 99.30 ± 2.88.

**Conclusions:**

The proposed method had high throughput as the analysis involved simple sample pre-treatment procedure and a relatively short run-time (< 15 min). The results demonstrated that the method would have a great value when it is applied in the therapeutic monitoring and pharmacokinetic studies for LND.

## Background

Multiple myeloma (MM) is a B-cell malignancy of the plasma cell and represents the second most common haematological malignancy (about 10%), with non-Hodgkin’s lymphoma being the most common. It is estimated that approximately 21,500 new cases of multiple myeloma are diagnosed per annum with approximately 16,000 deaths from the disease annually within the European Union [1]. Multiple myeloma is characterized by an asymptomatic or subclinical phase before diagnosis (possibly for several years), a chronic phase lasting several years and an aggressive terminal phase. Multiple myeloma is primarily a disease of the elderly, with a median age at diagnosis of 68 years [2]. The disease leads to progressive morbidity and eventual mortality by lowering resistance to infection and causing significant skeletal destruction (with bone pain, pathological fractures, and hypercalcaemia, anaemia, renal failure [3], and, less commonly, neurological complications and hyperviscosity. From the time of diagnosis, the survival without treatment is between 6 to 12 months and extends to 3 years with chemotherapy. Approximately 25% of patients survive 5 years or longer, with fewer than 5% surviving longer than 10 years. MM is characterized with the production of a homogeneous immunoglobulin fraction, called myeloma protein, by the malignant plasma cells [4]. The classical triad of symptoms is plasmacytosis (> 30% of plasma cells in the bone marrow), myeloma protein either in the urine or blood, and lytic bone lesions [4,5].

In the 1990s, thalidomide (Thalomid®, Celgene Corporation) was used empirically in treatment of MM based on its antiangiogenic activity and clinical activity in refractory or relapsed myeloma [6]. However, thalidomide has significant and dose-limiting side effects such as sleepiness, constipation, neuropathy and teratogenicity [7]. These toxic effects promoted the search for more potent but less toxic thalidomide derivatives [8]. Lenalidomide (LND) is a potent novel thalidomide analog which demonstrated remarkable clinical activity against myeloma cells [8-13], via a multiple-pathways mechanism [7,8,14-19].

LND has a more improved side effects profile than its parent compound thalidomide, nevertheless, it causes some dose-dependent side effects such as thrombocytopenia, venous thromboembolism, and myelosuppression [20,21]. These side effects can be managed by combination therapy and/or careful dose adjustment [22,23]. Because LND is primarily excreted via kidneys, patients with renal insufficiency or failure must be dose adjusted to prevent the exacerbation of its myelosuppressive effects [10,18]. Because of the structural relation of LND to thalidomide, a teratogenic effect can not ruled out, thus effective contraception must be used by female patients [24,25]. Furthermore, studies showed large inter-individual pharmacokinetic variability with concentration–toxicity relationship [26]. For these reasons, a risk management, monitoring blood counts, and therapeutic drug monitoring are required to achieve the highest therapeutic benefits of LND and prevent its fatal complications [9,27,28]. Nevertheless, the therapeutic profile of LND is anticipated to encourage the development of new pharmaceutical preparations for LND. As a consequence, there is an increasing demand for proper analytical technologies for determination of pharmacokinetic parameters in bioequivalence studies for LND, as well as in its therapeutic monitoring.

Extensive literature survey showed that there were only two methods for the determination of LND in plasma [29,30]. These two methods involved liquid chromatography-coupled with mass spectrometric detectors (LC-MS). These two methods offered adequate sensitivities; however they employed the expensive mass detectors that are not available in most laboratories, and involved laborious liquid-liquid sample extraction procedures that negatively affected the accuracy of the results, and limit the throughput of the procedures in screening of large number of specimens. Accordingly, the development of a new alternative analytical method for the determination of LND in plasma with adequate sensitivity, improved simplicity, lower cost, and higher throughput is urgently needed.

Fluorescence-based HPLC has been used as a sensitive and less costly alternative approach to LC-MS. For these reasons, the present research proposal was directed towards the development and validation of a new simple and sensitive HPLC method with fluorescence detection for the determination of LND in plasma samples. The method involved a very simple non-extractive isolation of LND from plasma samples using protein precipitation with acetonitrile followed by masking biogenic amines by treatment with copper acetate, and derivatization with fluorescamine (FLC). The method was successfully applied to determination of LND in spiked human plasma samples.

## Experimental

The experimental research that is reported in this manuscript did not get any approval of ethics committee as the research has not been carried out on humans or animals.

### Chemicals and materials

Lenalidomide (LND), Free Base (3-(4’ aminoisoindoline-1’-one)-1-piperidine-2, 6-dione) was purchased from LC Laboratories (Woburn, MA, USA), 165 New Boston Street Woburn, MA 01801, USA) and used as received. Fluorescamine was purchased from Sigma Chemical Co. (St. Louis, MO, USA). Copper acetate AR was obtained from BDH, Poole, UK. Human plasma samples were collected from normal healthy volunteers at King Khaled University Hospital (Riyadh, Saudi Arabia), and they were kept frozen at −20°C until the time of analysis. Acetonitrile, tetrahydrofuran and all the other solvents were of HPLC grade (Merck, Darmstadt, Germany). Water was Millipore filtered. All other materials were of analytical grade.

### Preparation of solutions

#### Lenalidomide (LND) standard solution

An accurately weighed amount of LND was quantitatively transferred into a calibrated volumetric flask, dissolved in methanol and completed to volume with the same solvent to produce a stock solution of 1 mg/mL and kept in the refrigerator. On the day of analysis, the stock solution was further diluted stepwise with water to obtain a working standard solution containing 1.0 μg/mL.

#### Fluorescamine solution

An accurately weighed amount (5 mg) of Fluorescamine was transferred into a 10-mL volumetric flask, dissolved in acetonitrile and completed to volume with the same solvent to produce a stock solution of 0.05% (w/v). The solution was freshly prepared and kept at -20°C pro-tected from light to be used within seven days.

#### Phosphate buffer solution

Weighed amounts of di-sodium hydrogen orthophosphate (5.04 g) and potassium di-hydrogen orthophosphate (3.01 g) were dissolved in about 700 mL distilled water. The pH of the solution was adjusted to 4.0±0.1 with glacial acetic acid using a calibrated pH-meter (Microprocessor pH meter BT-500, Boeco, Germany).

### Chromatographic system

HPLC apparatus consisted of a Shimadzu system (Shimadzu Corporation, Kyoto, Japan) equipped with two solvent delivery systems (LC-20 AD VP) with FCV-12AH high pressure flow channel changeover valve, SIL-20A auto-sampler, CTO-10A column oven, SPD-10A UV-visible detector,, RF-10A XL fluorescence detector, and SCL-10A vp system controller. The chromatographic separations were performed on an analytical column Hypersil BDS C18 (250 mm length × 4.6 mm i.d., 5 μm particle diameter) manufactured by Hypersil, ThermoQuest Corporation (England). The column temperature was kept constant at 25 ± 2°C. Separations were performed in isocratic mode. The mobile phase used for separation consisted of phosphate buffer (pH 4): methanol: tetrahydrofuran (70: 10: 20, v/v) pumped at flow rate of 1.0 mL/min. The mobile phase was filtered by a Millipore vacuum filtration system equipped with a 0.45 μm pore size filter, degassed by ultrasonication, and further by bubbling with helium gas. The samples (50 μL each) were injected by the aid of the auto-sampler. The fluorescence detector was set at 382 nm as an excitation wavelength and 495 nm as an emission wavelength. The system control and data acquisition were performed by Shimadzu CLASS-VP software, version 5.032 (Shimadzu Corporation, Kyoto, Japan). The relation between the area of LND peak and its concentration was used as the basis for the quantification. Alternatively, the regression equation was derived.

#### General procedure and construction of the calibration curve

Accurately measured aliquots of LND working stock solution (1.0 μg/mL) ranging from 20–400 μL were transferred into eight separate reaction vials each containing 500 μl plasma and 100 μl of a 0.2% solution of copper acetate. The volumes in all vials were adjusted, as necessary, to 1.0 mL with water. The vials were heated in a boiling water-bath for 15 min. and cooled. 1.0 mL of acetonitrile was added to each vial and centrifuged for 10 min at 10000 rpm. Portions of 500 μL of the supernatant solutions were transferred into each of a set of eight vials followed by 300 μL of water. 200 μL of FLC solution (0.05% w/v) was added to all vials. This resulted in a series of LND standard solutions covering the working range of 5–100 ng/mL in the final reaction mixture. The reaction in each vial was allowed to proceed at room temperature for 5 min. before injecting 50 μL into the HPLC system. Peak area values of the reaction product between LND and FLC obtained at retention time of around 11.6 min. were plotted versus the LND concentration. A reagent blank treated in the same manner but using water instead of the LND working stock solution was prepared. Also another blank was prepared omitting the addition of copper acetate.

## Results and discussion

### Design and strategy for assay development

LND contains a weakly absorbing chromphore in its molecule. This weak absorptivity does not facilitate the adequate sensitivity for determination of LND levels in plasma without pre-concentration of the samples, particularly at the initial and elimination pharmacokinetic phases. On the other hand, LND has no native fluorescence and therefore a pre-colmn derivatization procedure was necessary. Fluorescamine (4^′^-phenylspiro[2-benzofuran-3,2^′^-furan]-1,3^′^-dione) is a fluorogenic reagent which reacts almost instantaneously with a wide variety of nucleophiles including primary amines, even at very low concentrations, forming fluorescent pyrrolinone moieties. The reaction is almost instantaneous at room temperature in aqueous media. The products are highly fluorescent, whereas the reagent and its degradation products are nonfluorescent [31]. Fluorescamine does not react with secondary amines and forms a non-fluorescent adduct upon binding to free NH3. LND contains a primary aromatic amino group which makes it a good candidate readily reacting with fluorescamine giving a highly fluorescent adduct.

### Optimization of reaction variables

**Figure 1:**
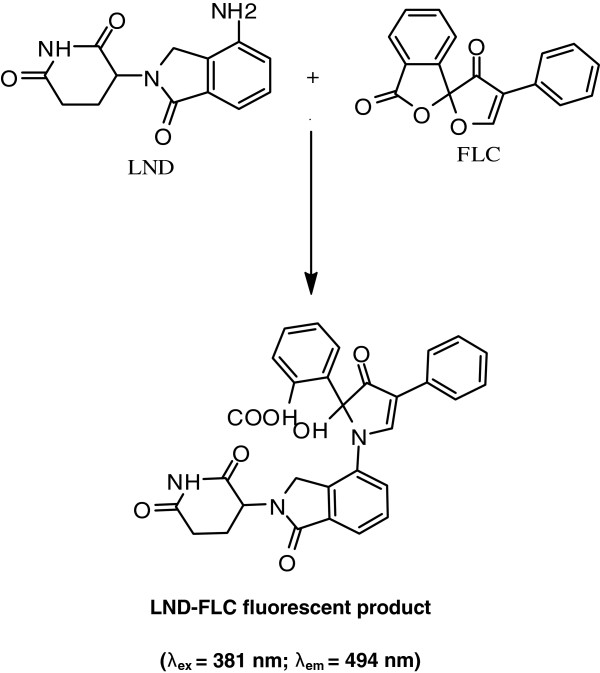
Scheme for the reaction pathway between LND and FLC.

From our previous study of the reaction between LND and FLC [32], we found that the reaction was dependent on FLC concentration, pH of the reaction medium, the nature of the diluting solvent and the duration of the reaction time. The investigations revealed that the best conditions for the reaction were found by maintaining a final concentration of 0.0025% w/v of FLC, a neutral pH using simple water as diluting solvent and leaving the reaction to proceed for five minutes at room temperature. Regarding the stoichiometry of the reaction and by analogy to our study mentioned above, the reaction is proposed to proceed as shown in Figure 1.

The excitation and emission spectra of the LND-FLC derivative formed under the optimized conditions were investigated using RF-5301 PC spectrofluorimeter (Shimadzu, Kyoto, Japan). The reaction product was found to be fluorescent showing the highest fluorescence intensity at λex of 382 nm and λem of 495 nm. The present study was devoted to adopt the above reaction in the developing a sensitive HPLC method with fluorescence detection for determination of LND in plasma.

### Method development

Plasma is highly rich in nitrogen containing compounds such as amino acids. Consequently, interference from such endogenous amino acids is obviously expected. In our present study, this interference was eliminated through a selective complex formation between those α-amino acids and copper ions furnished by addition of copper acetate [33], a modification from the method which describes the use copper hydroxide as the source of copper ions [34]. Copper acetate did not interfere with the determination of LND based on the following facts: (1) copper forms a stable complex with α-amino acids via binding of two amino acids by an amino nityrogen and a carboxylic oxygen (i.e. NNOO coordination bonding); (2) The presence of carboxylic group in α position to the primary amino group is essential for the formation of the complex; (3) LND contains a primary amino group however it does contain the carboxylic oxygen to form the copper complex. For these reasons, copper acetate did not interfere with the determination of LND in the method described herein. Acetonitrile is added to precipitate the large molecule plasma proteins which are then separated by centrifugation. The centrifugation also helps separation of the amino acid-copper complexes. The supernatant layers which contain the LND were transferred into the set of vials ready for the derivatization procedure as explained above.

### Method validation

#### Selectivity, linearity, limit of detection and limit of quantitation

**Figure 2:**
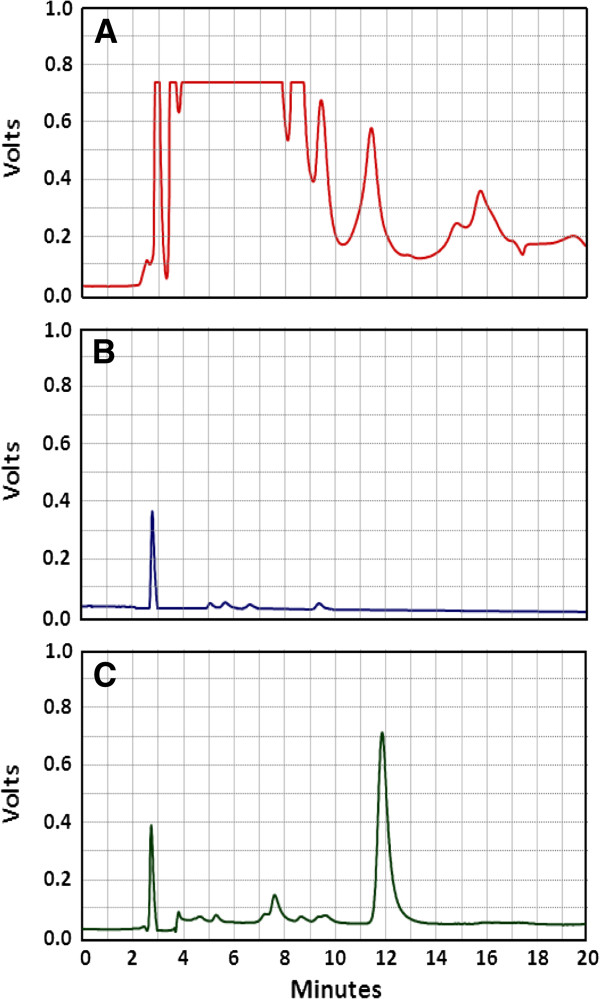
**Chromatogram obtained from FLC-derivatized blank plasma before treatment with copper acetate (A), after treatment with copper acetate (B), derivatization product of LND-spiked plasma (50 ng mL**^**−1**^**) pretreated with copper acetate (C).** The peak of retention time 11.6 min corresponds to LND-FLC fluorescent product.

The selectivity of the method was evaluated by carrying out blank experiments in the mobile phase to identify the reagent peaks and the peaks due to the derivatized plasma components. Typical chromatograms obtained from FLC-derivatized blank plasma before and after treatment with copper acetate are given in Figure 2A and Figure 2B, respectively. The chromatogram of the derivatization product between LND-spiked plasma (50 ng/mL) after treatment with copper acetate and FLC reagent is given in Figure 2C. The effect of treatment of the plasma with copper acetate before derivatization with FLC is clearly observed in from the chromatograms. The chromatogram given in Figure 2A shows how great is the interference from the endogenous amines if the plasma is not treated with copper acetate before derivatization procedure. Very high intensity peaks cover almost the whole range including the area where the derivatized LND peak appears (~ 11.6 min). On the other hand, the chromatogram of the plank plasma derivatized with FLC after pretreatment with copper acetate (Figure 2B) is absolutely free from any interference around the retention time of LND. These results demonstrated the great importance of treating the plasma samples with copper acetate before derivatization with FLC.

The Chromatographic parameters and performance data of the proposed HPLC method for determination of LND in plasma are presented in Table 1. Under the optimized conditions, a linear relationship with good correlation coefficient (r = 0.9993 ± 0.00027, n = 6) was found between the peak area of LND-FLC complex (Y) versus LND concentration (X) in the range of 5–100 ng/mL. The experiments were performed using eight-point standard series. The mean regression equation of the calibration curve obtained was Y = 1.27 × 10^5^ + 3.11 × 10^5^ X. The % RSD value for the slopes of the calibration curves was 1.89% (n = 6). The limit of detection (LOD) and limit of quantitation (LOQ) were calculated according to the ICH guidelines for validation of analytical procedure based on the standard deviation of the response and the slope of the calibration curve [35] using the formula: LOD or LOQ = κσ/S, where κ = 3.3 for LOD and 10 for LOQ, σ is the standard deviation of the response, and S is the slope of the calibration curve. Calculations on 6 replicate experimental injections, the LOD and LOQ were 0.8 and 2.3 ng/mL respectively, and the relative standard deviations (RSD) did not exceed 2%. The LOD achieved in this method was lower than that achieved in our previous method involving the same derivatization procedure [32]. This is attributed due to the difference in the detection systems between the conventional fluorimeter (in the previous study) and the fluorescence HPLC detector in the method described herein.

**Table 1:** Chromatographic parameters and performance data of the proposed HPLC method for determination of LND in plasma

**Parameter**	**Value**
Retention time for LND-FLC complex (min)	11.6
Retention time of the nearest adjacent peak (min)	9.1
Capacity factor (K’) for LND-FLC complex	8.4
Resolution between LND-FLC complex and the adjacent peak	3.5
LND-FLC complex peak asymmetry factor at 10% peak height	1.33
Number of theoretical plates (N) per meter	13764
Height equivalent to theoretical plate (μm)	72.6
Correlation coefficient	0.9995
Slope (b) ± SD	3.11 × 10^5^ ± 5.89 × 10^3^
Intercept (a) ± SD	1.272009× 10^5^ ± 7.7 × 10^4^
Linearity range (ng/mL)	5 – 100
LOD (ng/mL)	0.8
LOQ (ng/mL)	2.3

#### Accuracy and precision

The accuracy and precision of the proposed method was determined by intra-day and inter-day replicate analysis of plasma spiked with different concentrations of LND covering the working linear range. The inter-day assays were carried out on five different days at the same concentration levels for spiked plasma samples. The recovery values from the intra-day analysis were 97.93 -103.65% with a mean value of 100.85 ± 2.07 whereas the values for the inter-day analysis were 97.85 – 104.60 with a mean value of 100.91 ± 1.87 (Table 2), indicating the accuracy and precision of the proposed method.

**Table 2:** Intra-assay and inter-assay precision and accuracy for determination of LND in spiked human plasma

**Nominal conc. (ng/mL)**	**Intra-assay **^**a**^		**Inter-assay **^**b**^	
	**Measured conc. (ng/mL)**	Recovery (%) *	**Measured conc. (ng/mL)**	Recovery (%) *
5	4.935	99.70	5.230	104.60
10	9.793	97.93	10.080	100.80
20	20.66	103.30	19.810	99.05
30	30.399	101.33	30.621	102.07
40	41.103	102.76	40.380	100.95
50	51.823	103.65	50.610	101.22
75	73.96	98.61	75.578	100.77
100	99.494	99.49	97.85	97.85
Mean ± S.D.		100.85 ± 2.07		100.91 ±1.87

a : Average of three replicates.

b : Average of four replicates.

* : %RSD not exceeding 2%.

#### Robustness and ruggedness

The robustness of the method was evaluated by making minor changes on the parameters affecting the reaction between the analyte and the reagent in addition to the chromatographic parameters. In order to measure the extent, the most critical parameters were interchanged while keeping the other parameters unchanged. The chromatographic parameters were interchanged within the range of 1-10% of the optimum recommended conditions. The parameters involved were: the pH of the phosphate buffer used in the mobile phase, the ratio between the components of the mobile phase, pH of the derivatization reaction mixture, and column temperature. The chromatographic profile including: capacity factor (k’), retention time (Rt), peak asymmetry, resolution and column efficiency were calculated and compared with those of the system suitability (Table 1). The results revealed that the method was robust for these small changes in the parameters. However, increasing the pH value above 7.3 resulted in marked decrease in the detector signal. The increase in the column temperature generally decreases the k’ values, and the column temperature has to be maintained at 25±2°C.

The ruggedness of the method was evaluated by applying the recommended analytical procedures on the same HPLC system on different days on the analysis of series of LND-spiked plasma samples. The values of the capacity factor (k’), retention time (Rt) and peak areas obtained each time were not significant.

## Conclusions

A simple, accurate and precise HPLC method with fluorescence detector for trace determination of LND after its pre-column derivatization with FLC has been successfully developed and validated. The sample preparation procedure was very simple and robust as it did not involve any liquid-liquid extraction of the sample. It was based on selective complexation of the endogenous α- amino acids with copper ions and precipitation of the proteins with acetonitrile before the derivatization reaction. The derivatized sample was directly injected into the HPLC system. The chromatographic separation was based on a reversed phase mechanism carried out under isocratic elution mode for only less than 15-min total run time. The analytical results demonstrated that the proposed method is suitable for the accurate quantification of LND in human plasma at concentrations as low as 2.3 ng/mL, with a wide linear range. The simple procedure involved in the sample preparation and the short run-time added the property of a higher throughput to the method. It is valuable for the combined pharmacokinetic and bioavailability studies of LND in human subjects.

## Abbreviations

MM: Multiple myeloma; LND: Lenalidomide; FLC: Fluorescamine; HPLC: High-performance liquid chromatography; LOD: Limit of detection; LOQ: Limit of quantification; SD: Standard deviation; RSD: Relative standard deviation

## Competing interests

The authors declare that they have no competing interests.

## Authors’ contributions

NYK conducted the method development and validation. IAD proposed the subject, designed the study, participated in the results discussion and revised the manuscript. TAW participated in method development and validation. AAA participated in results discussion and writing the manuscript. All authors read and approved the final manuscript.
